# Factors associated with significant liver necroinflammation in chronic hepatitis B patients with cirrhosis

**DOI:** 10.1038/srep33093

**Published:** 2016-09-12

**Authors:** Sheng-Sen Chen, Kang-Kang Yu, Qing-Xia Ling, Chong Huang, Ning Li, Jian-Ming Zheng, Su-Xia Bao, Qi Cheng, Meng-Qi Zhu, Ming-Quan Chen

**Affiliations:** 1 Department of Infectious Diseases and Hepatology, Huashan Hospital, Fudan University, Shanghai 200040, China

## Abstract

We determined the association between various clinical parameters and significant liver necroinflammation in patients with chronic hepatitis B (CHB) related cirrhosis. Two hundred patients with CHB related cirrhosis were recruited in the final analysis. Clinical laboratory values and characteristics were obtained from the medical record. We performed analyses of the relationships between independent variables and significant liver necroinflammation by using binary logistic regression analysis and discriminant analysis. Significant liver necroinflammation (grade≥2) was found in 58.0% (80/138) of antiviral therapy patients and 48.4% (30/62) of non antiviral therapy patients respectively. Also, there were some significant differences in serum hepatitis B surface antigen (HBsAg), serum hepatitis B e antigen (HBeAg) and serum hepatitis B virus (HBV) DNA between antiviral therapy and non antiviral therapy patients. After that, aspartate aminotransferase (AST), total bilirubin (TBIL), total bile acid (TBA), prothrombin time (PT), aspartate aminotransferase to platelet ratio index (APRI) and serum HBV DNA were confirmed as independent predictors of significant liver necroinflammation in CHB patients with cirrhosis by univariate analysis and multivariate analysis (p = 0.002, 0.044, 0.001, 0.014, 0.01 and 0.02 respectively). Finally, receiver operating characteristic (ROC) curve analysis and discriminant analysis validated that these six variables together have strong predictive power to evaluate significant liver necroinflammation.

Chronic hepatitis B (CHB) represents a significant public health burden, approximately 400 million people are affected by hepatitis B virus (HBV) infection worldwide^1^. CHB related liver injury constitutes a major risk factor for development of end-stage liver disease including cirrhosis and hepatocellular carcinoma in HBV endemic region^2^,^3^. The annual incidence of cirrhosis among CHB patients was reported to be approximately 2.1–6.0%^4^. After advancement to cirrhosis, liver disease may continue to progress and decompensated complications can occur, especially in those with active HBV replication^5^. A substantial proportion of patients with cirrhosis still have active HBV replication, as evidenced by positive hepatitis B e antigen (HBeAg) or high HBV DNA levels^6^, which are the strongest factors for disease progression^7^,^8^.

Clinical studies to date have revealed that oral antivirals are generally safe and effective in HBV suppression, with an improvement in liver disease in patients presenting with HBV-related decompensation^4^. Recent studies also showed that profound viral suppression with newer, more potent antivirals showed short-term efficacy as assessed within 1 or 2 years^9^,^10^,^11^,^12^. Liver injury in chronic hepatitis B can be mitigated by current antiviral treatment, as evidenced by normalization of elevated serum alanine aminotransferase (ALT) and improvement of liver histology in treated patients^13^,^14^. The main objective of current antiviral therapy was to block the progression of chronic liver injury and reduce the portion of liver cirrhosis among the CHB patients^14^,^15^; however, between 10% to 37% of CHB patients with normal ALT already having significant necroinflammation and fibrosis^16^,^17^, thus for these CHB patients with normal ALT, liver cirrhosis may has been developed at the initial antiviral treatment, and severe liver necroinflammation in these patients who have developed liver cirrhosis whether can be mitigated by antiviral treatment still remains unknown. Hence the effect of antiviral therapy on liver necroinflammation in CHB patients with cirrhosis should be investigated.

In addition, liver histological changes (necroinflammation) of the CHB patients with persistent normal ALT and liver cirrhosis may only be confirmed by liver biopsies. Although liver biopsy remains an integral part in determining liver necroinflammation and fibrosis, it is an invasive procedure and patients may also come up some complications (subcapsular haematoma, haemothorax, haemobilia and shock) after liver biopsy^18^. Sampling error and intra-observer variations are also unavoidable^19^,^20^. Therefore, it is imperative to seek non-invasive factors associated with liver necroinflammation of CHB patients with cirrhosis. More importantly, these factors can be used to build a model for determining the significant liver necroinflammation and this model should be easy to practice at the bedside.

To address these issues above, we presented our analysis of liver histology in a large cohort of Chinese CHB patients with cirrhosis. The aim of this study was to evaluate the effect of antiviral therapy in this cohort and seek the factors can be easily used at the bedside to determine the significant liver necroinflammation in CHB patients with cirrhosis.

## Methods

### Patients

This retrospective cohort study was performed at Huashan Hospital, a tertiary hospital in Shanghai, China. We collected the data of all patients diagnosed as liver cirrhosis from the electronic medical record system. Then all cases who met the inclusion criteria below in our hospital from January 2008 to December 2013 were included in our study: (1) patients with persistent normal ALT (PNALT) or minimally elevated ALT. PNALT is defined by continually normal ALT levels tested at least on 3 occasions over a 1- year period prior to liver biopsy, whereas minimally elevated ALT levels are defined as ALT levels ranging between 1× upper limit of normal (ULN) and 2 × ULN^21^, and ULN of ALT is commonly considered as 40 U/L^22^. (2) Patients who were diagnosed as liver cirrhosis must be confirmed by liver biopsy, and liver cirrhosis considered by clinical, biochemical, and morphological criteria were excluded. (3) No hepatocellular carcinoma (HCC) and progressive extrahepatic malignancy. (4) Liver cirrhosis must be only caused by CHB, and decompensated liver disease, chronic hepatitis C or D virus infection, primary biliary cirrhosis, autoimmune hepatitis, Wilson’s disease, alcoholic liver disease, non-alcoholic fatty liver disease, drug-induced liver injury and HIV coinfection were excluded. (5) No virological events that can change the necroinflammation were existed in patients.

### Ethics statements

All data were anonymously analyzed without individual patient consent due to the retrospective nature of the study. This study protocol was approved by the institutional review boards at Fudan University and Huashan Hospital.

### Liver biopsy and histology

Liver biopsies were obtained using 16G biopsy needles and guided by ultrasonography. Qualified biopsy specimens were 1.5 cm to 1.8 cm in length, and 10 portal spaces were included in the specimens. Biopsies were fixed, paraffin-embedded, and two serial sections stained with hematoxylin-eosin-safran for morphological evaluation and Masson’s trichrome stain for assessment of fibrosis respectively. Scheuer’s scoring system^23^ was used to semi-quantify the histologic necroinflammation from G0 to G4 and fibrosis stages from S0 to S4 by the same pathologist, who was blinded to the biochemical and virologic results of the patients. Significant histological abnormality was defined as necroinflammation grade ≥G2 and/or fibrosis stage ≥S2^24^. Since cases included in the final study all were confirmed as liver cirrhosis by liver biopsy, we just recorded the histologic ecroinflammation (G0 to G4) at last.

### Data collection and abstraction

Data were abstracted and recorded in a standard form by two investigators and then reviewed in duplicate by another three investigators, all of whom accepted training to familiarize themselves with the performance of the data form at the commencement of the study. Serum biochemical tests were performed within 7 days prior to liver biopsy. The detailed data were recorded as follows: (1) general information (gender, age, occupation, height, weight, etc.); (2) diagnosis at admission and discharge, disease history (including history of allergies), HBV infection history of family, drinking history, antiviral therapy history (in this study, the time of antiviral treatment initiation must be earlier than liver biopsy and the time interval between liver biopsy and treatment initiation was at least 48 weeks; patients in treated group were given nucleoside analogues not the interferon, and the duration of antiviral therapy was approximately 1–2 years); (3) results of liver biopsy, including necroinflammation grade, hepatic tissue iron stain, hepatic tissue HBsAg, hepatic tissue HBcAg, and hepatic tissue HBV-DNA; (4) symptoms and signs; (5) results of biochemical examinations, including alanine aminotransferase (ALT), aspartate aminotransferase (AST), serum total bilirubin (TBIL), direct bilirubin (DBIL), albumin (ALB), globulin (GLB), cholinesterase (CHE), and total bile acid (TBA); CHE was measured with Ellman’s method^25^; (6) results of blood routine examination and coagulation function; (7) HBV serological markers, including hepatitis B surface antigen (HBsAg), hepatitis B e antigen (HBeAg), hepatitis B c antibody (HBcAb) and HBV-DNA; the serological HBV-DNA levels less than 2000 IU/ml means low viremia^26^, so we considered 2000 IU/ml as cutoff value; (8) abdomen ultrasound results (size of liver and spleen, especially splenomegaly), Poulin *et al*. defined splenomegaly as moderate if the largest dimension is 11–20 cm and severe if the largest dimension is greater than 20 cm^27^; (9) AST to platelet ratio index (APRI) was calculated according to the formula which was devised by Chun-Tao Wai^28^.

### Statistical analyses

Continuous variables were presented as mean ± standard deviation and Kruskal-Wallis test was used for comparison of non-parametric continuous variables. Categorical variables were expressed as frequency and percentage and analyzed by Fisher’s exact test. The correlation coefficients (r) were calculated using Spearman’s correlation. Multivariate logistic regression was used to determine the independent predictors of significant histological necroinflammation. A prediction model built by using significant variables obtained from multivariate logistic regression with P < 0.05. Then we measured the area under the curve (AUC) of the receiver operating characteristic (ROC) curve in order to validate the predictive power of the prediction model. The optimal cutoff value was determined to maximize the sum of sensitivity and specificity.

The discriminant analysis is a multivariate statistical method of classification, and the classification of a case is based on the combination of prior probabilities with discriminant functions. Therefore, the discriminant analysis was performed to further confirm the predictive efficiency of prediction model for significant histological necroinflammation in CHB patients with cirrhosis. All statistical tests were two-sided, and P values less than 0.05 were considered as statistically significant. The statistical analyses were performed by using SPSS version 21.0 and GraphPad Prism version 5.0.

## Results

### Study population

**Figure 1:**
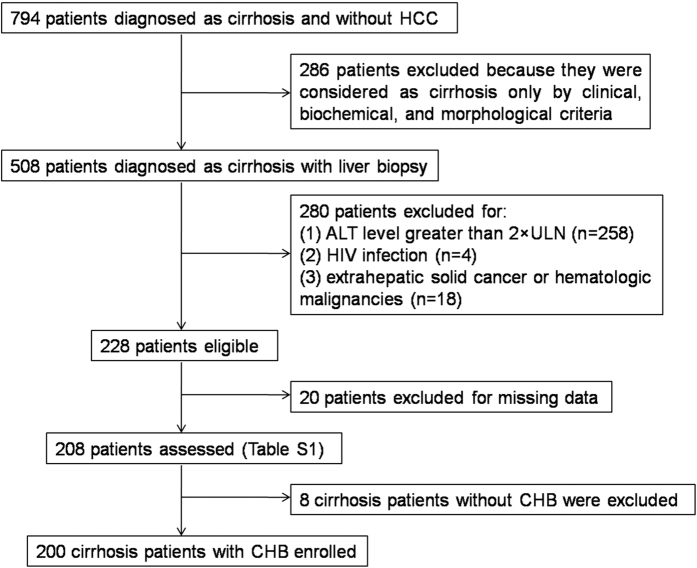
Flow diagram depicting the participants’ selection process.

During the study period, 794 patients with cirrhosis and without HCC were admitted, but 286 patients were first excluded because they were considered as cirrhosis only by clinical, biochemical, and morphological criteria. ALT levels of 258 patients greater than 2×ULN, 4 patients were HIV-infected, and 18 patients had extrahepatic solid cancer or hematologic malignancies, so these patients above all were excluded. Because of missing data, 20 additional patients were excluded. Then, 208 patients with cirrhosis were included and their characteristics are summarized in Table S1. However, among these 208 patients, liver cirrhosis of 8 patients were not caused by CHB, thus a total of 200 CHB patients with cirrhosis were finally included (Fig. 1).

Characteristics of the 200 study subjects are shown in Table 1. Of the enrolled patients, 138 patients had antiviral therapy (treated group) and 62 did not (untreated group). The treated group consisted of 82 males and 56 females (age, 43.5 ± 10.04 years), while the untreated group have 41males and 21 females (age, 43.2 ± 10.7 years). On the whole, the antiviral-treated group showed lower serum HBsAg level and lower HBV replication activity than the untreated group (Table 1). Compared to the untreated group, the proportion of serum HBeAg positive patients were significantly lower in treated group. In addition, baseline characteristics of the 200 patients can be seen in Table S2, which showed that characteristics at baseline between treated and untreated patients were almost no different except the serum HBeAg and HBV DNA.

### Correlation between serum HBV antigen (HBsAg and HBeAg) and HBV DNA

**Figure 2:**
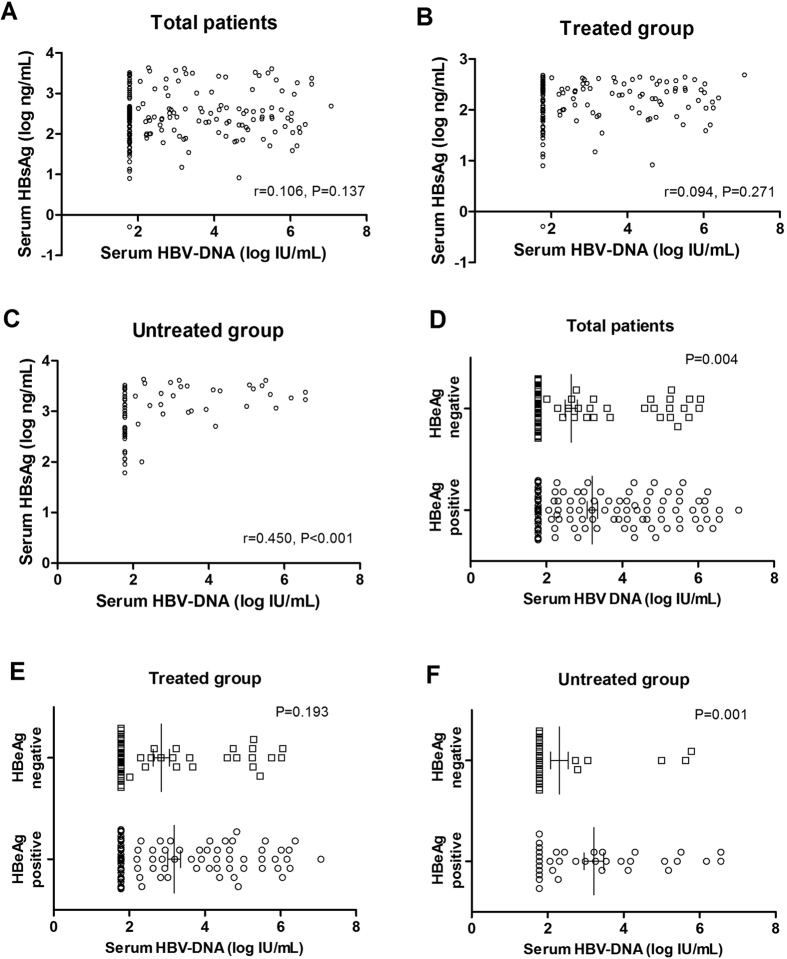
Correlation between serum HBsAg levels and serum HBV DNA levels in all patients (**A**) treated patients (**B**) and untreated patients (**C**); correlation between serum HBeAg and serum HBV DNA levels in total patients (**D**), treated patients (**E**) and untreated patients (**F**). The correlation coefficients (r) between HBsAg and HBV DNA were calculated using Spearman’s correlation; the relationship between serum HBeAg and serum HBV DNA was identified using Kruskal-Wallis test.

From Table 1, we got that antiviral treatment can reduce the serum HBsAg level and HBVvira load, and also facilitate the positive HBeAg become negative. Then in order to further confirm the expression of serum HBV DNA, HBsAg and HBeAg whether affected by antiviral treatment or not, the correlation analyses were made between HBsAg and HBV DNA and between HBeAg and HBV DNA. For serum HBsAg level and HBV viral load, their correlation was not found in the entire cohort (Fig. 2A) and treated group (Fig. 2B); while in untreated group, we found the correlation between serum HBsAg level and HBV viral load (Fig. 2C). Additionally, correlation between serum HBeAg and HBV DNA levels was significant in all patients (Fig. 2D) and untreated group (Fig. 2F); whereas in the treated group, we did not find any correlation between serum HBeAg and HBV DNA levels (Fig. 2E).

### Distribution of significant necroinflammation among CHB patients with cirrhosis

**Figure 3:**
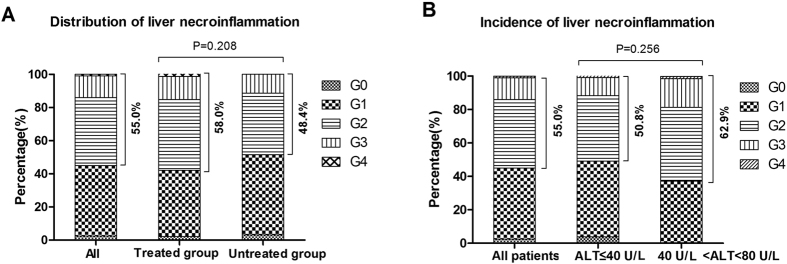
Distribution of liver necroinflammation among 200 chronic hepatitis B patients. (**A**) Distribution of liver necroinflammation in treated and untreated group; (**B**) Distribution of liver necroinflammation in groups with ALT ≤ 40 U/L and ALT 40–80 U/L. P values were calculated by the Fisher’s exact test.

The percentages of liver necroinflammation in all groups are shown in Fig. 3. Significant necroinflammation (≥G2) was found in 55.0% of all patients. Altogether 138 patients had antiviral therapy, of which 58.0% showed significant necroinflammation, while just 48.4% of untreated patients had significant necroinflammation (Fig. 3A), and the difference between these two groups did not find (P = 0.208). Also, among the 130 patients with ALT ≤ 40 U/ml, of which 50.8% showed significant necroinflammation, and in the group with minimally elevated ALT levels (40–80 U/ml), 62.9% patients showed significant necroinflammation (Fig. 3B). No difference about distribution of significant necroinflammation between group with ALT ≤ 40 U/ml and group with minimally elevated ALT levels (P = 0.256).

### Univariate and multivariate analysis of factors associated with liver necroinflammation

AS shown from Table 1 and Fig. 3A, no difference (P = 0.208) was found about the distribution of significant necroinflammation in treated group and untreated group. In addition, there was also no significant difference in necroinflammation (P = 0.256) occurred between group with ALT ≤ 40 U/ml and group with minimally elevated ALT levels (Fig. 3B). Since the variables above (antiviral treatment and ALT level) were not related with significant necroinflammation, subsequently, we added the variables and used univariate and multivariate analysis to seek the factors associated with significant liver necroinflammation in cirrhosis patients with CHB.

In univariate analysis, edema (P = 0.034), AST (0.003), CHE (0.024), TBA (0.040), serum HBeAg (0.043), serum HBV-DNA levels (0.001), and hepatic tissue iron stain (0.013) were factors associated with significant liver necroinflammation (Table 2). The multivariate analysis of clinical parameters independently associated with significant necroinflammation was also shown in Table 2. Higher AST level (OR = 1.137, P = 0.002), higher TBIL (OR = 1.07, P = 0.044), higher TBA (OR = 1.019, P = 0.001), higher PT (OR = 2.598, P = 0.014), higher APRI (OR = 1.581, P = 0.010) and higher serum HBV-DNA (OR = 1.968, P = 0.020) were independently correlated with significant necroinflammation in CHB patients with cirrhosis.

### Prediction model establishment and ROC curve analysis

**Figure 4:**
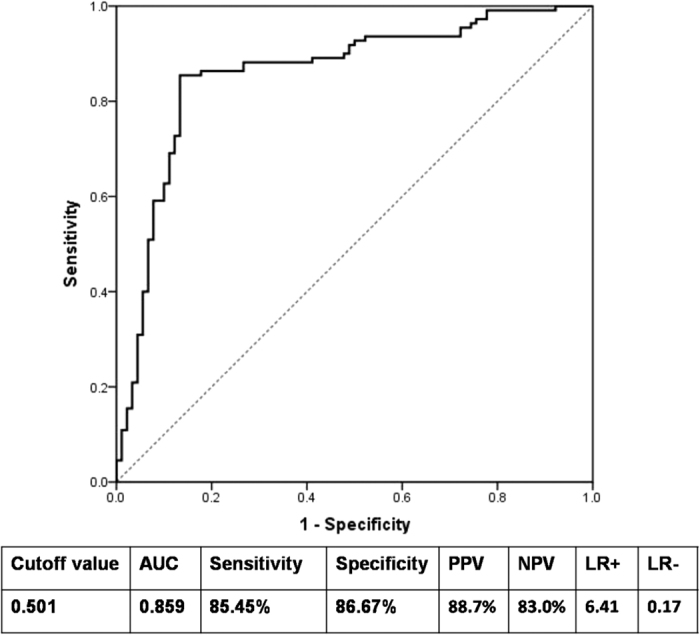
ROC curve for determining the predictive power of the prediction model including AST, TBIL, TBA, PT, APRI and serum HBV-DNA. PPV, positive predictive value; NPV, negative predictive value; LR+, positive likelihood ratio; LR−, negative likelihood ratio.

After that, the independent factors such as AST, TBIL, TBA, PT, APRI and HBV-DNA were together included in the multivariate logistic regression model again. Next we built a prediction model and got the prediction probability for significant necroinflammation in the cirrhosis patients with CHB (each patient had a prediction probability, the detailed can be seen from Table S3). Then we took the prediction probability as test variable and the actual classification of liver necroinflammation as state variable (≥G2 vs. <G2), and finally the ROC Curve was plotted by using SPSS 21.0 to determine predictive power of the model. As shown from the Fig. 4, the AUC of this model for predicting significant necroinflammation was 0.859, and optimal cutoff prediction probability was 0.501. Therefore, the CHB patient with cirrhosis whose prediction probability greater than 0.501 can be considered as significant necroinflammation according to the results of ROC curve (Fig. 4).

### Discriminant analysis for validating the predictive efficiency of prediction model

At last, the predictive power of prediction model included AST, TBIL, TBA, PT, APRI and HBV-DNA for discerning significant necroinflammation was also validated by discriminant analysis. It can be clearly seen from Table 3 that the overall predictive percentage was 89.5%. Also, more importantly, in the group with significant necroinflammation, it correctly classified 85.5% of the cases (Table 3). The discriminant analysis was made for each patient and the detailed information was shown in Table S4.

## Discussion

X. Du *et al*.^29^ identified that antiviral treatment could achieve significant histological improvement (necroinflammation and fibrosis) in CHB patients with cirrhosis, including those with persistently normal ALT. Nevertheless, in X. Du’s study, the CHB patients with cirrhosis just accounted for a small portion of all CHB patients; thus to what extent histological abnormalities (especially liver necroinflammation) in the CHB patients who had developed cirrhosis can be improved is still undecided. For the first time, the present study evaluated the effect of antiviral therapy on liver necroinflammation and other clinical features of CHB patients with normal ALT or ALT 1–2×ULN (all patients who had developed cirrhosis). We classified the CHB patients with cirrhosis into treated group and untreated group according to the patients whether given antiviral therapy or not (Some patients in the untreated group showed positive HBsAg, normal transaminases level and undetectable HBV-DNA, so these patients were not given antiviral treatment. However, the HBsAg positive cirrhotic patients with detectable HBV-DNA in the untreated group are not treated because of their low income. They cannot afford the long time antiviral treatment and refused this therapy), and analyzed the impact of antiviral therapy on liver necroinflammation and other clinical characteristics of CHB patients with cirrhosis. Then the results of the analyses revealed that the antiviral treatment can reduce serum HBsAg and serum HBV-DNA level (Table 1). Furthermore, compared to the untreated group, the number of patients with serum HBeAg positive was significantly smaller in treated group. Subsequently, for the purpose of further confirming serum HBsAg, HBeAg and HBV-DNA levels were influenced by antiviral therapy, we made the correlation analyses between HBsAg and HBV DNA and between HBeAg and HBV DNA respectively in treated and untreated patients (Fig. 2). In the correlation analyses, serum HBsAg correlated well with serum HBV DNA in patients without antiviral treatment (Fig. 2C), while in all patients and in patients with antiviral treatment, HBsAg had not any correlation with HBV DNA (Fig. 2A,B), indicating that HBsAg secretion or HBV DNA replication was affected by antiviral therapy. Further analyses revealed that the expression of HBeAg was relevant to HBV DNA levels in all patients and untreated group (Fig. 2D,F), whereas in treated group, there was no correlation between HBeAg and HBV DNA (Fig. 2E), which means that HBeAg seroconversion or HBV DNA replication was really influenced by antiviral treatment.

Currently, the measuring of ALT levels and liver biopsy are commonly used to assess liver damage and guide the initiation of antiviral therapy. According to the European Association for the Study of the Liver (EASL) and American Association for the Study of Liver Diseases (AASLD) guidelines recommendations for the treatment of chronic hepatitis B^15^,^30^, ALT >2 ULN is one of several indicators for the initiation of antiviral therapy. In the absence of moderate-to-severe necroinflammation or fibrosis observed on liver biopsy, treatment may not be initiated in patients with normal or minimally increased ALT. In addition, some data had indicated that a fair portion of patients with persistently normal ALT had significant necroinflammation or fibrosis^16^,^17^. Patients with normal or mildly increased ALT may have severe liver necroinflammation that should be recognized by liver biopsy. So for patients with normal or ≤2 ULN ALT and relatively high HBV DNA load, it may be challenging to determine whether antiviral therapy is required without liver biopsy.

Although liver biopsy is an accurate mean in determining liver necroinflammation and fibrosis, it is an invasive procedure and patients may also come up some complications (subcapsular haematoma, haemothorax, haemobilia and shock) after liver biopsy. Additionally, ALT alone is not sufficiently accurate to confirm the grading of liver necroinflammation^31^. Noninvasive methods such as FibroScan, transient elastography and acoustic radiation force impulse are increasingly being used as surrogates for biopsy to detect liver fibrosis^32^, and these methods may instruct clinicians regarding the initiation of antiviral therapy to an extent. However, these methods are incapable of evaluating liver necroinflammation. Therefore, it is an urgent need for seeking noninvasive factors to evaluate liver necroinflammation, especially the significant liver necroinflammation in cirrhosis patients with CHB.

As shown from Table 1 and Fig. 3A, approximately 55% (110/200) of patients with CHB-related cirrhosis and normal or minimally increased ALT levels had significant liver damage, and the liver necroinflammation was not affected by antiviral treatment (P = 0.208). We then continued to investigate whether there was any difference in liver necroinflammation in cirrhosis patients with different levels of ALT (ALT ≤ 1 ULN vs. 1 ULN <ALT<2 ULN). Our current study found that there was no significance difference in liver necroinflammation between groups of patients with ALT ≤ 1 ULN and ALT 1–2 ULN (P = 0.256) (Fig. 3B). Thereby, other factors associated with liver necroinflammation in CHB patients with cirrhosis should be further explored. Then relationships between other factors and significant liver necroinflammation were fuzzily confirmed by univariate analysis (Fisher’s exact test and Kruskal-Wallis test), the results was summarized in Table 2, which indicated that edema, AST, CHE, TBA, serum HBeAg, serum HBV-DNA and hepatic tissue iron stain were significantly correlated with liver necroinflammation grade ≥G2. Since univariate analysis could hardly manage the interference existed among these variables above, a multivariate analysis must be performed to identify the authenticity and validity of the factors detected from the univariate analysis. After that, a key finding in the multivariate logistic regression analysis was that AST, TBIL, TBA, PT, APRI and serum HBV-DNA are independent predictors for significant liver necroinflammation in cirrhosis patients with normal or mildly increased ALT levels (Table 2).

In order to identify the predictive power of AST, TBIL, TBA, PT, APRI and serum HBV-DNA for predicting significant liver necroinflammation, we developed a prediction model composed of the six variables by using multivariate logistic regression analysis again. The strong predictive power of the prediction model to evaluate significant liver necroinflammation can be described by the ROC curve (AUC = 0.859, P < 0.001) (Fig. 4). The cutoff value of prediction probability (0.501) was also determined by ROC curve. Notably, the patient with prediction probability greater than 0.501 was considered as significant liver necroinflammation. Ultimately, for the purpose of further validating the predictive efficiency of the prediction model including AST, TBIL, TBA, PT, APRI and serum HBV-DNA, a discriminant analysis was made for each patient enrolled in this study. The discriminant analysis made up of AST, TBIL, TBA, PT, APRI and serum HBV-DNA demonstrated a very strong overall predictive value for evaluating significant liver necroinflammation (overall predictive percentage: 89.5%). It is noteworthy that discriminant analysis showed a correct classification of 85.5% patients in the group with liver necroinflammation ≥G2 and 94.4% patients in the group with liver necroinflammation <G2 (Table 3). Based on the discriminant analysis, the posterior probability of liver necroinflammation ≥G2 resulted to range between 50% and 100% for all cases, few classification errors occurred when the posterior probability was higher than 80% (Table S4). Hence, the use of these six factors as a prediction model strengthens their importance as predictors for the liver necroinflammation, especially at higher grades. In addition, necroinflammation may not be an important parameter to decide treatment among CHB patients with cirrhosis; however, the necroinflammation status in the CHB patients can provide the information about the severity of chronic hepatitis with cirrhosis, and this information may be helpful for the acute hepatic failure prevention. Although applying parameters for necroinflammation observed in patients with cirrhosis to patients with chronic hepatitis without cirrhosis may be not appropriate, these factors confirmed in our study can provide a clue for seeking the parameters associated with necroinflammation in CHB patients without cirrhosis.

In conclusion, the expression of HBsAg, HBeAg and HBV-DNA in serum can be reduced by antiviral therapy in CHB patients with cirrhosis. However, liver necroinflammation of the patients included in the study was not affected by antiviral treatment. Furthermore, we found AST, TBIL, TBA, PT, APRI and serum HBV-DNA are predictive factors for liver necroinflammation, particularly in cirrhosis patients with normal (<1ULN) or mildly increased ALT (1–2ULN). More importantly, herein we were able to develop a prediction model that accurately predict liver necroinflammation of cirrhosis patients by using a panel of six variables (AST, TBIL, TBA, PT, APRI and serum HBV-DNA). Then ROC curve and discriminant analysis identified that this model has strong predictive power of evaluating significant liver necroinflammation in CHB patients with cirrhosis. Additionally, a prediction probability will be generated when the prediction model is applied in a CHB patient with cirrhosis, and the patient with prediction probability greater than 0.501 can be considered as significant liver necroinflammation. Finally, these results together implied that the prediction model including AST, TBIL, TBA, PT, APRI and serum HBV-DNA firstly built in our study can be an excellent tool to predict significant liver necroinflammation in CHB patients with cirrhosis.

## Additional Information

**How to cite this article**: Chen, S.-S. *et al*. Factors associated with significant liver necroinflammation in chronic hepatitis B patients with cirrhosis. *Sci. Rep.* **6**, 33093; doi: 10.1038/srep33093 (2016).

## Supplementary Material

Supplementary Information

## Figures and Tables

**Table 1:** Characteristics of the CHB related cirrhosis patients.

Characteristics	Treated group (n = 138)	Untreated group (n = 62)	P value*
Age (year), mean ± SD	43.5 ± 10.04	43.2 ± 10.7	0.846
Sex (male), n (%)	82 (59.4)	41 (66.1)	0.367
HBV infection history of family (yes), n (%)	46 (33.3)	18 (29.0)	0.624
Clinical Presentation, n (%)			
Haematemesis	24 (17.4)	15 (24.2)	0.334
Melena	17 (12.3)	13 (21.0)	0.135
Abdominal distension	81 (58.7)	39 (62.9)	0.641
Fever	2 (1.4)	0	0.854
Poor appetite	76 (55.1)	31 (50.0)	0.542
Fatigue	103 (74.6)	46 (74.2)	0.947
Gum bleeding	9 (6.5)	3 (4.8)	0.758
Oliguria	9 (6.5)	5 (8.1)	0.766
Dark urine	37 (26.8)	13 (21.0)	0.480
Edema	6 (4.3)	0	0.180
Biochemistry			
ALT (U/L)	40.15 ± 22.21	38.84 ± 38.87	0.109
AST (U/L)	51.16 ± 34.02	46.37 ± 43.44	0.401
TBIL (μmol/L)	26.66 ± 17.47	26.62 ± 13.86	0.764
DBIL (μmol/L)	8.93 ± 5.93	9.17 ± 5.71	0.635
ALB (g/L)	35.72 ± 5.30	36.26 ± 5.07	0.566
GLB (g/L)	30.27 ± 7.30	29.54 ± 5.65	0.550
CHE (U/L)	4027.25 ± 1268.72	4134.21 ± 1237.60	0.682
TBA (μmol/L)	50.27 ± 56.11	50.52 ± 44.49	0.943
Coagulation function			
INR	1.32 ± 0.60	1.35 ± 0.66	0.915
PT (s)	16.90 ± 5.98	16.60 ± 5.07	0.952
PTA (%)	75.77 ± 25.52	79.69 ± 33.10	0.688
APTT (s)	34.64 ± 6.22	37.75 ± 20.06	0.919
TT (s)	18.50 ± 5.77	18.13 ± 6.12	0.742
FIB (g/L)	2.19 ± 2.11	2.14 ± 0.58	0.306
Blood routine examination, mean ± SD			
WBC (10^9^/L)	2.58 ± 1.54	2.84 ± 2.76	0.621
RBC (10^12^/L)	3.73 ± 0.68	3.70 ± 0.58	0.686
HB (g/L)	105.17 ± 22.80	103.24 ± 20.37	0.431
PLT (10^9^/L)	50.18 ± 36.31	54.29 ± 43.01	0.686
APRI, mean ± SD	3.21 ± 2.74	3.42 ± 5.39	0.063
Serum HBsAg (ng/ml)	203.23 ± 135.56	1636.73 ± 1211.15	<0.001
Serum HBeAg (positive), n (%)	48 (34.8)	35 (56.5)	0.005
Serum HBcAb (positive), n (%)	136 (98.6)	58 (93.5)	0.076
Serum HBV-DNA (IU/ml), n (%)			0.002
≥2000	52 (37.7)	38 (61.3)	
<2000	86 (62.3)	24 (38.7)	
Hepatic tissue iron stain, n (%)			0.431
−	110 (79.7)	51 (82.3)	
+	21 (15.2)	8 (12.9)	
++	1 (0.7)	2 (3.2)	
+++	6 (4.3)	1 (1.6)	
Hepatic tissue HBsAg, n (%)			0.441
−	27 (19.6)	9 (14.5)	
+	55 (39.9)	27 (43.5)	
++	17 (12.3)	13 (21.0)	
+++	21 (15.2)	8 (12.9)	
++++	18 (13.0)	5 (8.1)	
Hepatic tissue HBcAg, n (%)			0.440
−	77 (55.8)	40 (64.5)	
+	48 (34.8)	21 (33.9)	
++	6 (4.3)	1 (1.6)	
+++	5 (3.6)	0	
++++	2 (1.4)	0	
Hepatic tissue HBV-DNA (positive), n (%)	68 (49.3)	26 (42.6)	0.442
Necroinflammation grade, n (%)			0.208
<G2	58 (42.0)	32 (51.6)	
≥G2	80 (58.0)	30 (48.4)	
Spleen volume (cm^3^), mean ± SD	1216.11 ± 569.59	1588.30 ± 793.50	0.069
Splenomegaly			0.763
Moderate	95 (68.8)	44 (71.0)	
Severe	43 (31.2)	18 (29.0)	

*P value: Categorical variables—Fisher’s exact test; Continuous variables—Kruskal-Wallis test

SD = standard deviation; s = second

ALT, alanine aminotransferase; AST, aspartate aminotransferase; TBIL, total bilirubin; DBIL, direct bilirubin;

ALB, albumin; GLB, globulin; CHE, cholinesterase; TBA, total bile acid; INR, International Normalized Ratio;

PT, prothrombin time; PTA, prothrombin time activity; APTT, activated partial thromboplastin time; TT, thrombin time;

FIB, fibrinogen; WBC, white blood cell; RBC, red blood cell; HB, haemoglobin; PLT, blood platelet count;

APRI, aspartate aminotransferase to platelet ratio index; HBV, hepatitis B virus; HBsAg, hepatitis B surface antigen; HBeAg, hepatitis B e antigen; HBcAb, hepatitis B c antibody; HBcAg, hepatitis B c antigen.

(−), negative; (+), 10% positive cells; (++), 11–50% positive cells; (+++), 51–80% positive cells; and (++++), more than 80% positive cells.

**Table 2:** Clinical parameters predictive of significant liver necroinflammation (grade≥2) by univariate and multivariate analysis.

Factor†	Univariate*	Multivariate‡	OR (95% CI)	P Value
	P Value	β		
Age (year) (continuous)	0.128	−0.025	0.975 (0.936–1.015)	0.223
Sex (male vs. female)	0.267	0.658	1.932 (0.695–5.371)	0.207
HBV infection history of family (yes vs. no)	0.879	0.533	1.705 (0.702–4.142)	0.239
Clinical Presentation (yes vs. no)				
Haematemesis	0.151	1.055	2.872 (0.916–8.250)	0.069
Melena	0.558	−0.423	0.655 (0.204–2.107)	0.478
Abdominal distension	0.774	0.566	1.762 (0.732–4.240)	0.206
Fever	0.201	0.832	2.577 (0.842–5.673)	0.763
Poor appetite	0.887	0.564	1.757 (0.650–4.754)	0.267
Fatigue	0.519	−1.015	0.363 (0.109–1.210)	0.099
Gum bleeding	0.069	−0.614	0.541 (0.067–4.383)	0.541
Oliguria	0.269	−1.312	0.269 (0.051–1.412)	0.121
Dark urine	0.627	0.352	1.423 (0.537–3.771)	0.479
Edema	**0.034**	−1.081	0.376 (0.128–1.369)	0.563
Biochemistry (continuous)				
ALT (U/L)	0.056	0.006	1.006 (0.986–1.026)	0.551
AST (U/L)	0.003	0.052	1.137 (1.065–1.653)	**0.002**
TBIL (μmol/L)	0.584	0.074	1.077 (1.002–1.157)	**0.044**
DBIL (μmol/L)	0.853	0.136	1.145 (0.977–1.344)	0.095
ALB (g/L)	0.051	−0.003	0.997 (0.909–1.093)	0.946
GLB (g/L)	0.179	−0.027	0.973 (0.904–1.047)	0.464
CHE (U/L)	**0.024**	0.013	1.114 (0.989–1.201)	0.623
TBA (μmol/L)	**0.040**	0.019	1.019 (1.007–1.132)	**0.001**
Coagulation function (continuous)				
INR	0.742	0.237	1.267 (0.755–2.128)	0.370
PT (s)	0.683	0.955	2.598 (1.217–5.546)	**0.014**
PTA (%)	0.780	−0.010	0.990 (0.956–1.026)	0.587
APTT (s)	0.538	0.034	1.034 (0.987–1.182)	0.152
TT (s)	0.269	0.048	1.049 (0.958–1.149)	0.299
FIB (g/L)	0.616	−0.224	0.799 (0.350–1.825)	0.595
Blood routine examination (continuous)				
WBC (10^9^/L)	0.788	−0.080	0.923 (0.775–1.109)	0.367
RBC (10^12^/L)	0.090	−0.574	0.563 (0.277–1.147)	0.114
HB (g/L)	0.411	−0.008	0.992 (0.971–1.013)	0.455
PLT (10^9^/L)	0.305	−0.003	0.997 (0.988–1.007)	0.549
APRI (continuous)	0.128	0.458	1.581 (1.117–2.237)	**0.010**
Serum HBsAg (ng/ml) (continuous)	0.185	0.013	1.024 (0.985–1.136)	0.702
Serum HBeAg (positive vs. negative)	**0.043**	0.378	1.460 (0.609–3.497)	0.397
Serum HBcAb (positive vs. negative)	0.803	0.879	2.410 (0.243–4.545)	0.452
Serum HBV-DNA (IU/ml) (continuous)	**0.001**	0.914	1.968 (1.125–5.362)	**0.020**
Hepatic tissue iron stain (positive vs. negative)	**0.013**	1.076	2.933 (0.988–8.696)	0.053
Hepatic tissue HBsAg (positive vs. negative)	0.356	0.608	1.838 (0.577–5.848)	0.303
Hepatic tissue HBcAg (positive vs. negative)	0.249	0.705	2.024 (0.802–5.102)	0.136
Hepatic tissue HBV-DNA (positive vs. negative)	0.120	0.704	2.023 (0.807–5.070)	0.133
Splenomegaly (severe vs. moderate)	0.284	0.342	1.408 (0.619–3.204)	0.415
Antiviral therapy (yes vs. no)	0.975	−0.195	0.823 (0.31–2.183)	0.695

ALT, alanine aminotransferase; AST, aspartate aminotransferase; TBIL, total bilirubin; DBIL, direct bilirubin; ALB, albumin; GLB, globulin; CHE, cholinesterase; TBA, total bile acid; INR, International Normalized Ratio; PT, prothrombin time; PTA, prothrombin time activity; APTT, activated partial thromboplastin time; TT, thrombin time;FIB, fibrinogen; WBC, white blood cell; RBC, red blood cell; HB, haemoglobin; PLT, blood platelet count; APRI, aspartate aminotransferase to platelet ratio index; HBV, hepatitis B virus; HBsAg, hepatitis B surface antigen; HBeAg, hepatitis B e antigen; HBcAb, hepatitis B c antibody; HBcAg, hepatitis B c antigen.

^*^Univariate analysis: Categorical variables—Fisher’s exact test; Continuous variables—Kruskal-Wallis test.

^‡^Multivariate analysis: Binary logistic regression analysis.

^†^For continuous variables, the odds ratio represents that the possibility of significant liver necroinflammation change n-fold with one unit.

**Table 3:** Classification table of discriminant analysis.

Actual liver necroinflammation grade	Group size (n)	Predicted liver necroinflammation grade		
		≥G2 (n)	<G2 (n)	Correct percentage
≥G2	110	94	16	85.5%
<G2	90	5	85	94.4%
Overall percentage				89.5%

Predictive power of AST, TBIL, TBA, PT, APRI, and serum HBV-DNA for predicting the liver necroinflammation grade. This procedure is designed to develop a set of discriminating functions which can help predict ≥G2 vs. <G2 based on the values of other quantitative variables; 200 cases were used to develop a model to discriminate between the ≥G2 vs. <G2; six predictor variables were entered. Amongst the 200 observations used to fit the model, 89.5% was correctly classified.
