# Supplementary material for: Factors associated with significant liver necroinflammation in chronic hepatitis B patients with cirrhosis
Source: Sci Rep. 2016 Sep 12;6:33093. doi: 10.1038/srep33093 (PMC5018887; doi:10.1038/srep33093)
Supplement: Supplementary Information [file srep33093-s1.pdf]

Factors associated with significant liver necroinflammation in chronic hepatitis B patients with cirrhosis

Sheng-Sen Chen[1], Kang-Kang Yu[1], Qing-Xia Ling[1], Chong Huang[1], Ning Li[1], Jian-Ming Zheng[1], Su-Xia Bao[1], Qi Cheng[1], Meng-Qi Zhu[1], Ming-Quan Chen[1 *]

1. Department of Infectious Diseases and Hepatology, Huashan Hospital, Fudan University, Shanghai 200040, China;

*****Correspondence and requests for materials should be addressed to M.-Q.C.(email: bagews@163.com; Tel: +86-21- 52887965; Fax: +86-21-62480170)

Table S1. **Demographics and clinical variables for 200 CHB related cirrhosis patients and 8 non-CHB related cirrhosis patients**

**Characteristics**	**Whole population (n=208)**	**CHB related cirrhosis (n=200)**	**Non-CHB related cirrhosis (n=8)**
Age (year), mean ± SD	43.40±10.37	43.39±10.24	43.88±14.22
Sex(male), n (%)	147(70.7)	145(72.5)	55(27.5)
HBV infection history of family (yes), n(%)	64(30.8)	64(32.0)	0
Clinical Presentation, n (%)			
Haematemesis	39(18.8)	39(19.5)	0
Melena	30(14.4)	30(15.0)	0
Abdominal distension	124(59.6)	120(60.0)	4(50.0)
Fever	2(1.0)	2(1.0)	0
Poor appetite	110(52.9)	107(53.5)	3(37.5)
Fatigue	156(75.0)	149(74.5)	7(87.5)
Gum bleeding	12(5.8)	12(6.0)	0
Oliguria	14(6.7)	14(7.0)	0
Dark urine	52(25.0)	50(25.0)	2(25.0)
Edema	7(3.4)	6(3.0)	1(12.5)
Antiviral therapy (yes), n(%)	138(66.3)	138(69.0)	0
Follow-up time(month), mean ± SD	38.23±10.15	38.09±10.23	41.84±7.75
Biochemistry			
ALT(U/L)	39.67±28.01	39.75±28.34	37.88±19.02
AST(U/L)	49.76±36.54	49.68±37.15	52.00±16.03
TBIL(μmol/L)	26.69±16.33	26.65±16.40	27.58±15.52
DBIL(μmol/L)	9.10±6.17	9.00±5.85	11.56±12.12
ALB(g/L)	35.80±5.29	35.89±5.22	33.61±6.78
GLB(g/L)	30.26±7.02	30.05±6.83	35.71±9.84
CHE(U/L)	4059.17±1285.28	4060.41±1257.03	4028.25±1982.17
TBA (μmol/L)	50.63±52.96	50.35±52.67	57.75±63.32
Coagulation function			
INR	1.32±0.61	1.33±0.62	1.12±0.21
PT(s)	16.72±5.64	16.81±5.71	14.49±2.88
PTA(%)	77.5±28.0	77.0±28.1	89.6±26.0

APTT(s)	35.57±12.12	35.61±12.33	34.56±4.40
TT(s)	18.44±6.23	18.38±5.87	19.84±12.88
FIB(g/L)	2.19±1.75	2.18±1.78	2.41±0.46
Blood routine examination			
WBC(10^9/L)	2.68±2.03	2.66±1.99	3.21±2.86
RBC（10^{12}/L）	3.64±0.66	3.65±0.68	3.60±0.77
HB（g/L）	101.98±22.40	102.09±22.50	99.13±20.91
PLT(10^9/L)	51.30±37.83	51.46±38.45	47.50±16.60
APRI, mean ± SD	3.29±3.73	3.28±3.76	3.56±3.11
Serum HBsAg(ng/ml)	198.90±163.73	205.60±162.61	0.12±0.08
Serum HBeAg(positive), n(%)	125(60.1)	125(62.5)	0
Serum HBcAb(positive), n(%)	196(94.2)	194(97.0)	0
HBV-DNA(copies/ml), n(%)			
$>10^6$	24(11.5)	24(12.0)	0
10^5–10^6	25(12.0)	25(12.5)	0
10^4–10^5	15(7.2)	15(7.5)	0
10^3–10^4	29(13.9)	29(14.5)	0
$<10^3$	115(55.3)	107(53.5)	8(100.0)
Hepatic tissue iron stain, n(%)			
-	166(79.8)	161(80.5)	5(62.5)
+	32(15.4)	29(14.5)	3(37.5)
++	3(1.4)	3(1.5)	0
+++	7(3.4)	7(3.5)	0
Hepatic tissue HBsAg, n(%)			
-	21(10.1)	13(6.5)	8(100.0%)
+	70(33.7)	70(35.0)	0
++	46(22.1)	46(23.0)	0
+++	42(20.2)	42(21.0)	0
++++	29(13.9)	29(14.5)	0
Hepatic tissue HBcAg, n(%)			
-	123(59.1)	117(58.5)	6(75.0)
+	71(34.1)	69(34.5)	2(25.0)
++	7(3.4)	7(3.5)	0

+++	5(2.4)	5(2.5)	0
++++	2(1.0)	2(1.0)	0
Hepatic tissue HBV-DNA(positive), n(%)	94(45.4)	94(47.2)	0
Necroinflammation grade，n(%)			
G0	6(2.9)	5(2.5)	1(12.5)
G1	90(43.3)	85(42.5)	5(62.5)
G2	84(40.4)	82(41.0)	2(25.0)
G3	26(12.5)	26(13.0)	0(0)
G4	2(1.0)	2(1.0)	0(0)
Spleen size(cm), mean ± SD			
Length	17.86±3.13	17.88±3.11	17.50±3.96
Width	12.12±2.45	12.15±2.48	11.50±1.60
Thickness	5.92±1.20	5.94±1.21	5.50±1.07

**Table S2.** **Baseline characteristics of treated and untreated patients**			
**Characteristics**	**Treated group(n=138)**	**Untreated group(n=62)**	**P value***
Age (year), mean ± SD	42.3±9.01	41.2±11.6	0.746
Sex (male), n (%)	82(59.4)	41(66.1)	0.367
HBV infection history of family (yes), n(%)	46(33.3)	18(29.0)	0.624
Clinical Presentation, n (%)			
Poor appetite /Abdominal distension	68(49.3)	31(50.0)	0.924
Fever	15(10.9)	6(9.7)	0.799
Fatigue	57(41.3)	23(37.1)	0.641
Dark urine	55(39.9)	25(40.3)	0.950
Biochemistry			
ALT(U/L)	45.95±19.94	43.86±13.81	0.517
AST(U/L)	124.24±57.06	118.09±66.17	0.601
TBIL(μmol/L)	36.17±15.42	36.31±11.53	0.821
DBIL(μmol/L)	10.11±6.51	9.98±7.02	0.715
ALB(g/L)	32.17±4.36	33.42±6.11	0.611
GLB(g/L)	28.31±5.43	27.98±4.79	0.596
Coagulation function			
INR	1.46±0.81	1.51±0.93	0.793
PT(s)	14.19±4.86	13.99±5.13	0.822
PTA(%)	73.16±19.43	72.96±17.14	0.699
APTT(s)	35.83±5.19	36.17±9.04	0.402
Blood routine examination, mean ± SD			
WBC(10^9/L)	6.20±3.08	5.44±1.64	0.193
PLT(10^9/L)	169.13±42.16	165.25±49.27	0.593
Serum HBsAg(ng/ml)	1675.16±121.43	1659.43±176.15	0.632
Serum HBeAg(positive), n(%)	100(72.5)	33(53.2)	**0.008**
Serum HBV-DNA(log IU/ml)	5.67±2.317	3.48±1.96	**<0.001**

*****P value: Categorical variables——Fisher’s exact test; Continuous variables——Kruskal-Wallis test

SD= standard deviation; s= second

ALT, alanine aminotransferase; AST, aspartate aminotransferase; TBIL, total bilirubin; DBIL, direct bilirubin;

ALB, albumin; GLB, globulin; INR, International Normalized Ratio; PT, prothrombin time; PTA, prothrombin time activity; APTT, activated partial thromboplastin time; WBC, white blood cell; PLT, blood platelet count;

HBsAg, hepatitis B surface antigen; HBeAg, hepatitis B e antigen.

The data of treated and untreated patients at baseline were collected before antiviral treatment initiation.

**Table S3. Prediction probabilities list of prediction model for the significant liver necroinflammation(≥G2) in CHB patients with cirrhosis**			
**Case**	**Prediction probability**	**Predicted group**	**Actual group**
1	0.36558	0	0
2	0.58323	1	1
3	0.72338	1	1
4	0.45703	0	0
5	0.3272	0	1
6	0.50145	1	1
7	0.52855	1	1
8	0.48041	0	0
9	0.49406	0	0
10	0.62896	1	1
11	0.1356	0	0
12	0.79758	1	1
13	0.35076	0	0
14	0.32542	0	1
15	0.25537	0	0
16	0.44925	0	0
17	0.91739	1	1
18	0.34114	0	1
19	0.35494	0	0
20	0.42461	0	0
21	0.40858	0	0
22	0.39532	0	0
23	0.87312	1	1
24	0.95582	1	1
25	0.92701	1	1
26	0.52684	1	1
27	0.83338	1	1
28	0.50392	1	1
29	0.67874	1	1
30	0.46972	0	0
31	0.52901	1	0
32	0.5468	1	1
33	0.59522	1	1
34	0.64444	1	1
35	0.27997	0	1
36	0.99373	1	1
37	0.79518	1	1
38	0.96287	1	0
39	0.48916	0	1
40	0.61992	1	1
41	0.62632	1	1
42	0.17968	0	1
43	0.08887	0	0

44	0.23511	0	0
45	0.60157	1	1
46	0.90271	1	1
47	0.3056	0	1
48	0.10753	0	0
49	0.5067	1	1
50	0.40699	0	0
51	0.47979	0	0
52	0.80285	1	0
53	0.40161	0	1
54	0.55587	1	1
55	0.32642	0	0
56	0.36801	0	0
57	0.37538	0	0
58	0.13663	0	0
59	0.43707	0	0
60	0.65757	1	1
61	0.32743	0	0
62	0.36824	0	0
63	0.51556	1	1
64	0.46077	0	0
65	0.93678	1	1
66	0.48872	0	0
67	0.57588	1	1
68	0.39847	0	0
69	0.38724	0	0
70	0.99997	1	1
71	0.35141	0	0
72	0.43731	0	0
73	0.44886	0	0
74	0.22745	0	0
75	0.73725	1	1
76	0.34816	0	0
77	0.58372	1	1
78	0.88485	1	1
79	0.63112	1	1
80	0.68528	1	1
81	0.72053	1	1
82	0.35988	0	0
83	0.76058	1	1
84	0.50658	1	1
85	0.56616	1	1
86	0.51133	1	1
87	0.56087	1	1
88	0.78839	1	1
89	0.49789	0	0
90	0.36432	0	0

91	0.37686	0	1
92	0.64751	1	1
93	0.58447	1	1
94	0.21306	0	0
95	0.83375	1	1
96	0.53927	1	1
97	0.08979	0	0
98	0.4058	0	0
99	0.39547	0	1
100	0.4684	0	1
101	0.65904	1	0
102	0.38588	0	0
103	0.6457	1	1
104	0.61991	1	1
105	0.5649	1	1
106	0.65668	1	1
107	0.77978	1	1
108	0.58769	1	1
109	0.75759	1	1
110	0.72544	1	1
111	0.68044	1	1
112	0.7127	1	1
113	0.99297	1	1
114	0.39541	0	1
115	0.71334	1	1
116	0.31803	0	0
117	0.5661	1	1
118	0.51249	1	1
119	0.6359	1	1
120	0.64149	1	1
121	0.65932	1	1
122	0.43386	0	0
123	0.52188	1	1
124	0.5171	1	1
125	0.62016	1	1
126	0.5108	1	1
127	0.59103	1	0
128	0.71352	1	1
129	0.31638	0	0
130	0.66748	1	1
131	0.3929	0	1
132	0.82301	1	1
133	0.78328	1	1
134	0.42183	0	1
135	0.24279	0	0
136	0.42437	0	0
137	0.50816	1	1

138	0.65416	1	1
139	0.48285	0	0
140	0.71972	1	1
141	0.19194	0	0
142	0.42504	0	0
143	0.60081	1	1
144	0.26645	0	0
145	0.19723	0	0
146	0.96872	1	1
147	0.583	1	0
148	0.26623	0	0
149	0.36977	0	0
150	0.47384	0	0
151	0.54646	1	0
152	0.47112	0	0
153	0.77333	1	0
154	0.54535	1	1
155	0.14839	0	0
156	0.34807	0	1
157	0.23808	0	0
158	0.6739	1	1
159	0.35922	0	0
160	0.58841	1	0
161	0.99163	1	1
162	0.46755	0	1
163	0.60505	1	1
164	0.35932	0	0
165	0.52671	1	1
166	0.62546	1	1
167	0.71372	1	0
168	0.54559	1	1
169	0.78628	1	1
170	0.53753	1	1
171	0.61951	1	1
172	0.80357	1	1
173	0.7663	1	1
174	0.72714	1	1
175	0.68528	1	1
176	0.17436	0	0
177	0.41469	0	0
178	0.45933	0	0
179	0.73954	1	1
180	0.49823	0	0
181	0.37009	0	0
182	0.44294	0	0
183	0.87385	1	0
184	0.36402	0	0

185	0.76062	1	1
186	0.62126	1	0
187	0.35744	0	0
188	0.41006	0	0
189	0.26113	0	0
190	0.21969	0	0
191	0.34873	0	0
192	0.40546	0	0
193	0.46953	0	0
194	0.4905	0	0
195	0.60671	1	1
196	0.36458	0	0
197	0.2486	0	0
198	0.91484	1	1
199	0.4368	0	0
200	0.33711	0	0
0= Liver necroinflammation grade <G2, 1= Liver necroinflammation grade ≥G2			

Table S4: Probabilities list of discriminant analysis for the significant liver necroinflammation(G≥2) in patients with CHB related cirrhosis

Case	Actual Group	Highest Group					Second Highest Group			Discriminant Scores
		Predicted Group	P(D>d | G=g)		P(G=g | D=d)	Squared Distance	Group	P(G=g | D=d)	Squared Distance	Function 1
			p	df						
1	0	0	0.331	1	0.986	0.945	1	0.014	9.480	-2.131
2	1	1	0.595	1	0.750	0.283	0	0.250	2.480	0.416
3	1	0**	0.351	1	0.563	0.871	1	0.437	1.376	-0.225
4	0	0	0.679	1	0.794	0.171	1	0.206	2.866	-0.745
5	1	1	0.524	1	0.706	0.406	0	0.294	2.159	0.311
6	1	0**	0.411	1	0.619	0.676	1	0.381	1.649	-0.336
7	1	1	0.504	1	0.692	0.447	0	0.308	2.069	0.280
8	0	0	0.530	1	0.710	0.395	1	0.290	2.184	-0.530
9	0	0	0.451	1	0.652	0.569	1	0.348	1.829	-0.404
10	1	1	0.912	1	0.879	0.012	0	0.121	3.983	0.837
11	0	0	0.998	1	0.903	0.000	1	0.097	4.451	-1.162
12	1	1	0.150	1	0.995	2.076	0	0.005	12.584	2.389
13	0	0	0.572	1	0.968	0.319	1	0.032	7.134	-1.723
14	1	0**	0.401	1	0.610	0.707	1	0.390	1.603	-0.318
15	0	0	0.149	1	0.995	2.080	1	0.005	12.594	-2.601
16	0	0	0.732	1	0.817	0.117	1	0.183	3.114	-0.817
17	1	1	0.595	1	0.750	0.282	0	0.250	2.482	0.417
18	1	1	0.456	1	0.657	0.555	0	0.343	1.855	0.203
19	0	0	0.599	1	0.965	0.277	1	0.035	6.930	-1.685
20	0	0	0.908	1	0.878	0.013	1	0.122	3.966	-1.043
21	0	0	0.974	1	0.908	0.001	1	0.092	4.575	-1.191
22	0	0	0.877	1	0.927	0.024	1	0.073	5.112	-1.313
23	1	1	0.491	1	0.683	0.474	0	0.317	2.010	0.259
24	1	1	0.004	1	1.000	8.392	0	0.000	25.035	3.845
25	1	1	0.008	1	1.000	6.926	0	0.000	22.452	3.580
26	1	1	0.293	1	0.501	1.104	0	0.499	1.115	-0.103
27	1	1	0.077	1	0.997	3.134	0	0.003	15.029	2.718
28	1	0**	0.398	1	0.608	0.714	1	0.392	1.592	-0.314
29	1	1	0.728	1	0.950	0.121	0	0.050	6.021	1.295
30	0	0	0.916	1	0.880	0.011	1	0.120	4.003	-1.053
31	0	1**	0.716	1	0.810	0.133	0	0.190	3.036	0.584
32	1	1	0.386	1	0.597	0.752	0	0.403	1.537	0.081
33	1	1	0.686	1	0.797	0.163	0	0.203	2.900	0.544
34	1	1	0.975	1	0.908	0.001	0	0.092	4.572	0.979
35	1	1	0.384	1	0.595	0.759	0	0.405	1.526	0.077
36	1	1	0.001	1	1.000	10.535	0	0.000	28.648	4.194
37	1	1	0.156	1	0.995	2.013	0	0.005	12.427	2.367
38	0	0	0.866	1	0.866	0.029	1	0.134	3.754	-0.990
39	1	1	0.001	1	1.000	10.661	0	0.000	28.855	4.213

40	1	1	0.610	1	0.758	0.260	0	0.242	2.548	0.438
41	1	1	0.892	1	0.874	0.018	0	0.126	3.886	0.813
42	1	1	0.164	1	0.994	1.937	0	0.006	12.237	2.340
43	0	1**	0.257	1	0.990	1.285	0	0.010	10.500	2.082
44	0	0	0.444	1	0.979	0.586	1	0.021	8.249	-1.924
45	1	1	0.717	1	0.811	0.132	0	0.189	3.040	0.585
46	1	1	0.016	1	0.999	5.799	0	0.001	20.383	3.356
47	1	1	0.525	1	0.972	0.405	0	0.028	7.523	1.584
48	0	0	0.735	1	0.949	0.115	1	0.051	5.978	-1.497
49	1	0**	0.384	1	0.595	0.758	1	0.405	1.528	-0.288
50	0	0	0.963	1	0.910	0.002	1	0.090	4.638	-1.206
51	0	0	0.533	1	0.712	0.388	1	0.288	2.201	-0.536
52	0	0	0.925	1	0.883	0.009	1	0.117	4.051	-1.065
53	1	0**	0.376	1	0.588	0.783	1	0.412	1.493	-0.274
54	1	1	0.433	1	0.638	0.614	0	0.362	1.751	0.164
55	0	0	0.430	1	0.980	0.622	1	0.020	8.381	-1.947
56	0	0	0.685	1	0.956	0.165	1	0.044	6.311	-1.564
57	0	0	0.735	1	0.949	0.114	1	0.051	5.975	-1.496
58	0	0	0.404	1	0.613	0.698	1	0.387	1.617	-0.323
59	0	0	0.818	1	0.850	0.053	1	0.150	3.522	-0.929
60	1	1	0.396	1	0.606	0.721	0	0.394	1.582	0.099
61	0	0	0.436	1	0.979	0.607	1	0.021	8.327	-1.938
62	0	0	0.687	1	0.956	0.163	1	0.044	6.301	-1.562
63	1	0**	0.341	1	0.553	0.907	1	0.447	1.333	-0.206
64	0	0	0.654	1	0.782	0.201	1	0.218	2.751	-0.711
65	1	1	0.006	1	1.000	7.408	0	0.000	23.312	3.670
66	0	0	0.481	1	0.676	0.497	1	0.324	1.964	-0.453
67	1	1	0.549	1	0.722	0.359	0	0.278	2.272	0.349
68	0	0	0.900	1	0.923	0.016	1	0.077	4.982	-1.284
69	0	0	0.819	1	0.937	0.052	1	0.063	5.454	-1.387
70	1	1	0.001	1	1.000	10.911	0	0.000	29.266	4.251
71	0	0	0.577	1	0.968	0.312	1	0.032	7.102	-1.717
72	0	0	0.816	1	0.849	0.054	1	0.151	3.514	-0.927
73	0	0	0.735	1	0.818	0.114	1	0.182	3.127	-0.820
74	0	0	0.883	1	0.926	0.022	1	0.074	5.079	-1.306
75	1	1	0.376	1	0.983	0.784	0	0.017	8.953	1.834
76	0	0	0.556	1	0.969	0.346	1	0.031	7.263	-1.747
77	1	1	0.598	1	0.752	0.278	0	0.248	2.494	0.421
78	1	1	0.025	1	0.999	5.035	0	0.001	18.926	3.192
79	1	1	0.927	1	0.884	0.008	0	0.116	4.062	0.857
80	1	1	0.684	1	0.956	0.166	0	0.044	6.320	1.355
81	1	1	0.464	1	0.977	0.535	0	0.023	8.056	1.680
82	0	0	0.631	1	0.962	0.231	1	0.038	6.693	-1.639
83	1	1	0.374	1	0.585	0.791	0	0.415	1.481	0.058
84	1	0**	0.385	1	0.596	0.756	1	0.404	1.530	-0.289
85	1	1	0.575	1	0.739	0.314	0	0.261	2.391	0.388

86	1	0**	0.361	1	0.573	0.834	1	0.427	1.424	-0.245
87	1	1	0.461	1	0.660	0.544	0	0.340	1.874	0.210
88	1	1	0.175	1	0.994	1.839	0	0.006	11.991	2.304
89	0	0	0.430	1	0.635	0.623	1	0.365	1.735	-0.369
90	0	0	0.660	1	0.959	0.193	1	0.041	6.483	-1.598
91	1	0**	0.354	1	0.567	0.857	1	0.433	1.394	-0.233
92	1	1	0.952	1	0.913	0.004	0	0.087	4.693	1.008
93	1	1	0.603	1	0.754	0.271	0	0.246	2.516	0.428
94	0	0	0.651	1	0.780	0.205	1	0.220	2.735	-0.706
95	1	1	0.481	1	0.676	0.496	0	0.324	1.966	0.244
96	1	1	0.349	1	0.561	0.877	0	0.439	1.370	0.012
97	0	0	0.307	1	0.988	1.043	1	0.012	9.784	-2.180
98	0	0	0.954	1	0.912	0.003	1	0.088	4.685	-1.217
99	1	1	0.697	1	0.802	0.152	0	0.198	2.947	0.558
100	1	1	0.328	1	0.540	0.955	0	0.460	1.275	-0.029
101	0	0	0.905	1	0.877	0.014	1	0.123	3.949	-1.039
102	0	0	0.809	1	0.939	0.058	1	0.061	5.512	-1.400
103	1	1	0.966	1	0.910	0.002	0	0.090	4.621	0.991
104	1	1	0.846	1	0.859	0.038	0	0.141	3.657	0.754
105	1	1	0.484	1	0.678	0.491	0	0.322	1.977	0.248
106	1	1	0.885	1	0.926	0.021	0	0.074	5.066	1.092
107	1	1	0.202	1	0.993	1.631	0	0.007	11.448	2.225
108	1	1	0.624	1	0.766	0.241	0	0.234	2.611	0.457
109	1	1	0.283	1	0.989	1.151	0	0.011	10.108	2.021
110	1	1	0.437	1	0.979	0.604	0	0.021	8.314	1.725
111	1	1	0.717	1	0.952	0.132	0	0.048	6.098	1.311
112	1	1	0.509	1	0.974	0.435	0	0.026	7.652	1.608
113	1	1	0.001	1	1.000	10.489	0	0.000	28.573	4.187
114	1	0**	0.368	1	0.580	0.810	1	0.420	1.456	-0.259
115	1	1	0.506	1	0.974	0.443	0	0.026	7.685	1.614
116	0	0	0.548	1	0.970	0.361	1	0.030	7.331	-1.760
117	1	1	0.491	1	0.683	0.475	0	0.317	2.008	0.259
118	1	0**	0.356	1	0.568	0.854	1	0.432	1.399	-0.235
119	1	1	0.319	1	0.529	0.995	0	0.471	1.230	-0.050
120	1	1	0.997	1	0.903	0.000	0	0.097	4.456	0.952
121	1	1	0.866	1	0.929	0.028	0	0.071	5.176	1.116
122	0	0	0.841	1	0.858	0.040	1	0.142	3.634	-0.958
123	1	0**	0.312	1	0.523	1.021	1	0.477	1.202	-0.148
124	1	0**	0.334	1	0.546	0.934	1	0.454	1.300	-0.192
125	1	1	0.504	1	0.692	0.446	0	0.308	2.070	0.280
126	1	0**	0.364	1	0.576	0.825	1	0.424	1.436	-0.250
127	0	0	0.563	1	0.731	0.335	1	0.269	2.334	-0.580
128	1	1	0.505	1	0.974	0.445	0	0.026	7.694	1.615
129	0	0	0.378	1	0.983	0.776	1	0.017	8.924	-2.039
130	1	1	0.808	1	0.939	0.059	0	0.061	5.523	1.191
131	1	1	0.527	1	0.708	0.400	0	0.292	2.173	0.316

132	1	1	0.666	1	0.788	0.186	0	0.212	2.807	0.517
133	1	1	0.579	1	0.741	0.308	0	0.259	2.406	0.393
134	1	1	0.507	1	0.974	0.441	0	0.026	7.677	1.612
135	0	0	0.812	1	0.938	0.056	1	0.062	5.495	-1.396
136	0	0	0.910	1	0.879	0.013	1	0.121	3.975	-1.046
137	1	1	0.381	1	0.592	0.767	0	0.408	1.515	0.072
138	1	1	0.904	1	0.922	0.015	0	0.078	4.962	1.069
139	0	0	0.515	1	0.700	0.424	1	0.300	2.118	-0.508
140	1	1	0.469	1	0.977	0.525	0	0.023	8.014	1.672
141	0	0	0.976	1	0.896	0.001	1	0.104	4.314	-1.129
142	0	1^{**}	0.475	1	0.672	0.509	0	0.328	1.940	0.234
143	1	1	0.711	1	0.808	0.137	0	0.192	3.016	0.578
144	0	0	0.418	1	0.981	0.657	1	0.019	8.509	-1.969
145	0	0	0.838	1	0.857	0.042	1	0.143	3.616	-0.954
146	1	1	0.325	1	0.536	0.970	0	0.464	1.258	-0.037
147	0	0	0.788	1	0.942	0.073	1	0.058	5.645	-1.428
148	0	0	0.377	1	0.588	0.782	1	0.412	1.494	-0.274
149	0	0	0.697	1	0.954	0.152	1	0.046	6.230	-1.548
150	0	0	0.535	1	0.971	0.386	1	0.029	7.440	-1.780
151	0	0	0.690	1	0.799	0.159	1	0.201	2.915	-0.759
152	0	0	0.904	1	0.877	0.015	1	0.123	3.944	-1.038
153	0	0	0.354	1	0.985	0.859	1	0.015	9.202	-2.085
154	1	1	0.379	1	0.590	0.775	0	0.410	1.504	0.068
155	0	0	0.525	1	0.972	0.405	1	0.028	7.524	-1.795
156	1	1	0.738	1	0.949	0.112	0	0.051	5.959	1.282
157	0	1^{**}	0.314	1	0.524	1.015	0	0.476	1.208	-0.059
158	1	1	0.762	1	0.946	0.092	0	0.054	5.804	1.251
159	0	0	0.627	1	0.962	0.237	1	0.038	6.724	-1.645
160	0	0	0.588	1	0.746	0.294	1	0.254	2.448	-0.617
161	1	0^{**}	0.340	1	0.552	0.911	1	0.448	1.328	-0.204
162	1	1	0.585	1	0.744	0.299	0	0.256	2.433	0.401
163	1	1	0.485	1	0.678	0.488	0	0.322	1.982	0.249
164	0	0	0.627	1	0.962	0.236	1	0.038	6.720	-1.644
165	1	1	0.087	1	0.997	2.920	0	0.003	14.557	2.657
166	1	1	0.886	1	0.872	0.020	0	0.128	3.855	0.805
167	0	0	0.649	1	0.960	0.207	1	0.040	6.564	-1.614
168	1	1	0.380	1	0.591	0.771	0	0.409	1.509	0.070
169	1	1	0.666	1	0.788	0.186	0	0.212	2.806	0.517
170	1	1	0.341	1	0.553	0.907	0	0.447	1.332	-0.004
171	1	1	0.843	1	0.858	0.039	0	0.142	3.643	0.750
172	1	1	0.135	1	0.995	2.238	0	0.005	12.978	2.444
173	1	1	0.553	1	0.725	0.352	0	0.275	2.289	0.354
174	1	1	0.459	1	0.659	0.549	0	0.341	1.865	0.207
175	1	1	0.389	1	0.600	0.741	0	0.400	1.551	0.087
176	0	0	0.722	1	0.951	0.127	1	0.049	6.066	-1.515
177	0	0	0.981	1	0.897	0.001	1	0.103	4.338	-1.135

178	0	0	0.664	1	0.786	0.189	1	0.214	2.795	-0.724
179	1	1	0.427	1	0.633	0.630	0	0.367	1.724	0.154
180	0	0	0.428	1	0.634	0.628	1	0.366	1.726	-0.366
181	0	0	0.699	1	0.954	0.149	1	0.046	6.216	-1.545
182	0	0	0.777	1	0.835	0.081	1	0.165	3.322	-0.875
183	0	1**	0.388	1	0.599	0.746	0	0.401	1.545	0.084
184	0	0	0.658	1	0.959	0.196	1	0.041	6.497	-1.601
185	1	1	0.271	1	0.989	1.211	0	0.011	10.286	2.049
186	0	0	0.387	1	0.598	0.748	1	0.402	1.542	-0.294
187	0	0	0.615	1	0.964	0.253	1	0.036	6.810	-1.662
188	0	0	0.985	1	0.905	0.000	1	0.095	4.517	-1.177
189	0	0	0.305	1	0.988	1.052	1	0.012	9.810	-2.184
190	0	0	0.651	1	0.960	0.205	1	0.040	6.551	-1.612
191	0	0	0.560	1	0.969	0.340	1	0.031	7.234	-1.742
192	0	0	0.474	1	0.977	0.513	1	0.023	7.967	-1.875
193	0	0	0.597	1	0.751	0.279	1	0.249	2.490	-0.630
194	0	0	0.471	1	0.668	0.521	1	0.332	1.918	-0.437
195	1	1	0.752	1	0.825	0.100	0	0.175	3.207	0.632
196	0	0	0.662	1	0.959	0.191	1	0.041	6.471	-1.596
197	0	0	0.380	1	0.983	0.769	1	0.017	8.903	-2.036
198	1	1	0.012	1	0.999	6.349	0	0.001	21.403	3.468
199	0	0	0.820	1	0.851	0.052	1	0.149	3.532	-0.931
200	0	0	0.740	1	0.820	0.111	1	0.180	3.148	-0.826

**. Misclassified case ;   0= <G2, 1= ≥G2

The classification table lists the two highest groups amongst the classification functions for each of the 200 observations used to fit the model, as well as for any new observations. P (G | D) in the discriminant analysis was identified as posterior probability.
